# Treatment Nonadherence among Multimorbid Chronic Disease Patients: Evidence from 3515 Subjects in Indonesia

**DOI:** 10.3390/medicina60040634

**Published:** 2024-04-15

**Authors:** Ivan Surya Pradipta, Kevin Aprilio, Yozi Fiedya Ningsih, Mochammad Andhika Aji Pratama, Sofa Dewi Alfian, Rizky Abdulah

**Affiliations:** 1 Department of Pharmacology and Clinical Pharmacy, Faculty of Pharmacy, Universitas Padjadjaran, Sumedang 45363, Indonesia; 2 Drug Utilization and Pharmacoepidemiology Research Group, Center of Excellence in Higher Education for Pharmaceutical Care Innovation, Universitas Padjadjaran, Sumedang 45363, Indonesia

**Keywords:** multimorbidity, health determinants, epidemiological factors, disease management, treatment adherence and compliance

## Abstract

*Background and Objectives*: Multimorbid patients require intensive treatment for their diseases. However, little research has been given to their treatment adherence as part of its management. This study aims to determine the prevalence and characteristics of chronic disease multimorbidity in Indonesia, alongside its treatment nonadherence. *Materials and Methods*: We conducted a cross-sectional study using the fifth Indonesian Family Life Survey database among adult subjects aged ≥ 15 years with multimorbidity. Our descriptive and multivariate analyses include sex, age, formal education, ethnicity, geographic residence, demographic residence, household size, insurance ownership, annual income, current self-perceived health status, missing active days, smoking behavior, and body mass index. *Results*: We identified 3515 multimorbid patients, constituting 30.8% prevalence across chronic disease patients. Hypertension was found to be a prevalent component of multimorbidity (61.2%), followed by digestive diseases (44.5%) and arthritis (30.3%). We identified that 36.4% of the subjects were nonadherent to their chronic disease treatment. Characteristics associated with nonadherence were found to be a good self-perception of health (aOR 1.79, 95% CI 1.54–2.08), active smoking behavior (aOR 1.51, 95% CI 1.14–1.99), no smoking behavior (aOR 1.44, 95% CI 1.08–1.90), missing seven active/productive days or less in the past month due to poor health (aOR 1.36, 95% CI 1.10–1.68), no insurance ownership (aOR 1.20, 95% CI 1.04–1.39), age of 15–65 years (aOR 1.25, 95% CI 1.01–1.55), income below IDR 40 million (aOR 1.23, 95% CI 1.04–1.46), and household size of 2–6 people (aOR 1.17, 95% CI 1.01–1.36). *Conclusions*: While the prevalence of multimorbidity in Indonesia is generally similar to that observed in previous studies, we have identified patient characteristics related to nonadherence. We suggest that patient’s nonadherence was primarily dictated by their self-perception of health and treatment complexity. With the longstanding issue of nonadherence, this study indicated the need to consider creating patient-tailored treatment programs in clinical practice to improve adherence by considering individual patients’ characteristics.

## 1. Introduction

Improvements in healthcare quality and changes in environmental exposure have led to a global epidemiological shift from infectious diseases to noncommunicable diseases [1,2]. This shift has been most prevalent in low- and middle-income countries, such as Indonesia, over the past decade [1], where noncommunicable diseases such as chronic diseases have increased in prevalence and impact [2,3]. Inevitably, the prevalence of multimorbidity is also expected to increase compared to previous findings [4].

The rise of multimorbidity prevalence has led to issues in its treatment process, both for the patients and the healthcare system [5]. Multimorbid patients are at a higher risk of incurring complications [6], polypharmacy [7,8], decreased physical and mental functions [9,10], and a decreased quality of life [11,12] compared to those with only a single chronic disease. A study in Indonesia also reported a higher rate of inpatient and outpatient visits, catastrophic health expenditures, and a loss of productivity among multimorbid patients [13]. Along with the individual economic strains, due to the out-of-pocket expenses of its treatment [14,15,16], multimorbidity has also become an increasing burden for the healthcare system [17], as is occurring in Indonesia [16,18].

One of the critical aspects of chronic disease management is patient treatment adherence [19], with several studies indicating the additional health and economic burden caused by nonadherence [20,21]. However, treatment adherence was found to be lower in multimorbid patients compared to their single morbidity counterparts [22,23]; attributing the nonadherence primarily to the complexity and duration of treatment regimens as one of the clinical factors [24]. Nevertheless, several other systemic factors such as access to healthcare professionals and medical facilities have also been identified [25].

Further interventions are needed in formulating programs to improve the treatment adherence of multimorbid patients [1]. While several dimensions have been identified to contribute to treatment adherence, including socioeconomic-, health system-, therapy-, condition-, and patient-related factors [19], much of the subsequent research is focused solely on patients with a single chronic disease [26]. Existing research in Indonesia has, furthermore, only identified factors related to multimorbidity rather than its treatment nonadherence [4,18], limiting the development of adherence interventions for multimorbid patients [1].

Therefore, we formulate this study to identify the prevalence and characteristics related to multimorbidity and its treatment nonadherence in Indonesia. Through this study, we attempt to provide relevant clinical data for subsequent interventions in multimorbidity treatment.

## 2. Materials and Methods

### 2.1. Study Design and Participants

This study utilized a secondary database provided by the fifth Indonesian Family Life Survey (IFLS-5). IFLS-5, itself, is part of the ongoing IFLS project, which has been collecting longitudinal individual, household, and community data since 1993 [27], with IFLS-5 being the latest iteration of the survey conducted from 2014 to 2015 [28]. IFLS-5 was conducted by RAND Labor and Population and Universitas Gadjah Mada [29]. IFLS-5 employed multistage stratified sampling within 13 Indonesian provinces and their administrative subdivisions, constituting 321 enumeration areas. The sampling of 20 urban households and 30 rural households in each enumeration area represents approximately 83% of the Indonesian population [28].

We conducted a cross-sectional analysis of the IFLS-5 data among adult subjects aged ≥ 15 years, who reported having multimorbidities. Pediatric populations aged < 15 years were excluded from the study to better represent the association between each characteristic and nonadherence, noting the difference in measurements (Book V) and the reliance of the said population on their guardians for treatment. Multimorbidity among adult subjects was measured using the question *“Has a doctor/paramedic/nurse/midwife ever told you that you had [types of chronic disease]?”* (Book IIIB/CD05), based on a predetermined list of chronic diseases (Table S1) [29]. Subjects who reported having two or more chronic diseases were considered multimorbid. Subjects with missing outcome data were omitted from subsequent analysis processes.

### 2.2. Variables and Measures

We analyzed the socioeconomic-, disease-, and patient-related variables available in the IFLS-5 database, based on the treatment adherence framework established by the World Health Organization [19]. Sociodemographic-related variables include sex, age, formal education, ethnicity, geographic residence, demographic residence, household size, insurance ownership, and annual income. Patient-related variables include current self-perceived health status, future self-perceived health status in one year, missing active days, and depressive symptoms [30]. Disease-related variables include smoking behavior and body mass index (BMI) [31].

We set treatment adherence as the outcome of this study, where nonadherence was defined as subjects taking no prescribed treatments for any of their conditions. Treatment adherence is measured using the question *“Are you now taking [modes of treatment] to treat [types of chronic diseases] and their complications?”* (Book IIIB/CD09a) [29]. Subjects who reported taking no treatments for any of the chronic diseases were considered nonadherent. Further details on operational definitions of each variable are available in Table S1.

### 2.3. Data Analysis

We conducted a descriptive analysis to obtain the proportion of the subject’s multimorbidity, nonadherence, and characteristics. We also conducted regression analyses to determine the association of each characteristic to treatment nonadherence. Subjects with missing data for outcome variables were omitted from subsequent analyses via listwise deletion, whereas exposure variables exhibiting missing data of <10% were analyzed through the pairwise deletion of the missing exposure data [32].

Bivariate logistic analyses were conducted on each variable to select potential variables and eliminate multicollinearity for the subsequent multivariate logistic analysis, based on the bivariate *p*-value of <0.25. The following multivariate logistic analysis utilized an enter step for all selected variables, where its association with nonadherence is determined using a multivariate *p*-value of <0.05, with its odds ratio (OR) and 95% confidence interval (95% CI) used to determine the degree of such an association.

All statistical analyses in this study were conducted with SPSS^®^ Statistics for Windows™ version 22 from IBM^®^ Corporation, Armonk, NY, USA [33]. To promote the transparency of this study, we further reported this study following the STROBE (Strengthening the Reporting of Observational Studies in Epidemiology) statement guidelines for cross-sectional studies [34], as further stated in Table S2.

## 3. Results

**Figure 1:**
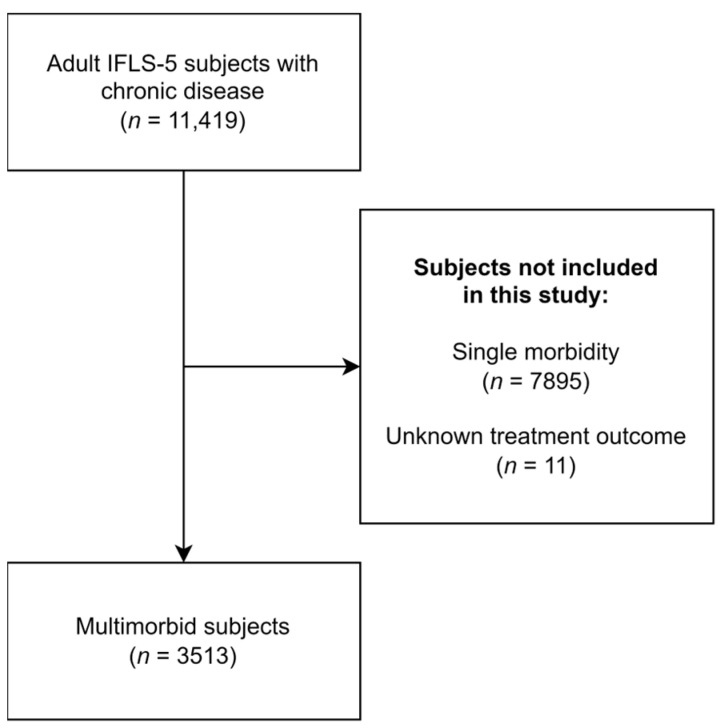
Flow diagram of subject selection.

Among 11,419 subjects with chronic diseases, 7895 of them had no multimorbidity and 11 of them had missing data. We analyzed 3513 subjects who met the inclusion criteria of this study, which indicated a multimorbidity prevalence of approximately 30.8% among patients with chronic diseases. This subject selection process is illustrated in Figure 1.

The first descriptive analysis of chronic diseases in the population indicated the prevalence of hypertension as a component of the subject’s multimorbidity. Following hypertension (61.2%), we also found digestive diseases (44.5%) and arthritis (30.3%) to be commonplace in constituting the subject’s multimorbidity. The lowest number of chronic diseases constituting multimorbidity were found to be psychiatric diseases (1.1%), memory-related diseases (1.5%), and cancer or other malignancies (3.0%). Further data regarding the prevalence of several chronic disease combinations are shown in Table 1.

**Figure 2:**
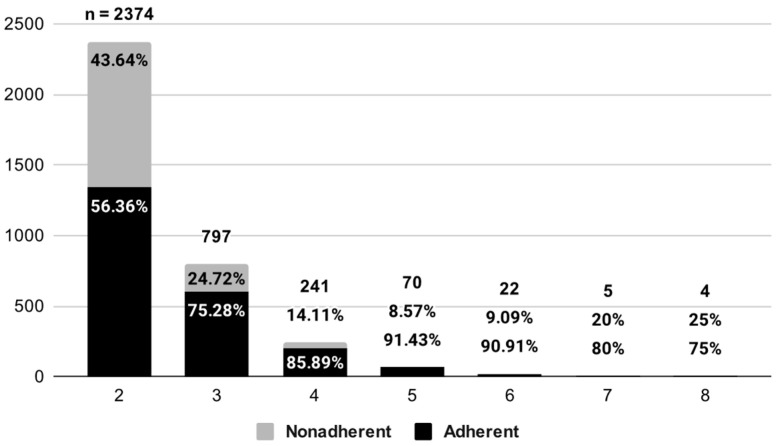
Distribution of treatment adherence across numbers of morbidity.

The second descriptive analysis of treatment nonadherence found that 36.4% of multimorbid subjects were nonadherent to their treatment. As illustrated in Figure 2, the prevalence of nonadherence across the numbers of morbidity by ratio followed a bell distribution curve, with the highest rate of adherence in patients with five to six morbidities and the lowest in patients with two morbidities. The highest number of morbidities among the subjects was found to be eight morbidities.

The third descriptive analysis of subjects’ characteristics indicated that the majority of multimorbid patients have sociodemographic-related characteristics of having attended formal education (92%), being aged 15–65 years (81%), residing in urban areas (66%), and being female (62%). Economically, 40% of the subjects were not working and 51% had an annual income of less than IDR 40 million. Patient-related characteristics indicate that 82% of the subjects had missing active/productive days of ≤7 days and 50% had a good self-perceived health status. Disease-related characteristics indicate that 68% were nonsmokers and 39% had an ideal BMI of between 18.5 and 24.9 kg/m^2^ [31]. However, several variables exhibit missing data above the acceptable range, i.e., depressive symptoms and health expectations, and were excluded in the following regression analyses [32]. These data are further shown in Table 2.

Upon subsequent regression analysis, we found associations between subjects who had a good self-perception of health (aOR 1.79, 95% CI 1.54–2.08), were an active smoker (aOR 1.51, 95% CI 1.14–1.99) or not a smoker (aOR 1.44, 95% CI 1.08–1.90), had monthly missing active days of 7 days or less (aOR 1.36, 95% CI 1.10–1.68), were aged between 15 and 65 years old (aOR 1.25, 95% CI 1.01–1.55), had an annual income of below IDR 40 million (aOR 1.23, 95% CI 1.04–1.46), and had a household size between 2 and 6 (aOR 1.17, 95% CI 1.00–1.36) with treatment nonadherence. All factors included in this study were found to have at least some of their components contributing to nonadherence. These data are presented in Table 3.

## 4. Discussion

The variables found in each factor of the treatment adherence factor confirm the multifaceted nature of treatment adherence, particularly among multimorbid patients [19]. In general, we found no significant differences in our findings of prevalence and characteristics compared to previous studies in Indonesia and other countries. However, characteristics associated with treatment nonadherence among multimorbid patients may indicate potential interventions to improve the treatment outcome. Subsequent parts of this discussion were divided into two subchapters, aspects related to the prevalence of multimorbidity itself and aspects related to multimorbidity treatment nonadherence.

### 4.1. Multimorbidity Prevalence

When compared to a previous study in Indonesia using IFLS-4 data gathered between 2007 and 2008 [4], several differences can be observed in the prevalence of multimorbidity. While the number of patients with chronic diseases has increased from 9438 in IFLS-4 (2007/2008) to 11,419 in IFLS-5 (2014/2015), the prevalence of multimorbidity among chronic disease patients has decreased from 48.8% in IFLS-4 to 30.8% in IFLS-5 [4]. In contrast, another study that presented the rate of multimorbidity through national health insurance claims (2015/2016) found multimorbidity to constitute 43.3% of chronic disease patients [18]. We believe that the differing data across studies may indicate the underreporting of chronic diseases [35,36], which may be attributed to the patient’s knowledge of their diseases [37].

The prevalence of multimorbidity in our findings generally aligned with the global average found in two meta-analyses, i.e., 33.1% and 37.2% [38,39]. We also noticed similar trends regarding the prevalence of multimorbidity among the sexes, where women are more likely to have multimorbidity, albeit with a smaller margin [38]. Our data also generally suggested that demographic differences, i.e., the differences between individuals, may be more associated with multimorbidity in the national scope [39]. Taking into account the small margin of multimorbidity prevalence across countries and their income status [38,39], we believe that geographic variances between countries are less associated with the incidence of multimorbidity compared to individual variances.

While our findings reported a lower number of morbidities associated with complications of uncontrolled hypertension compared to that from IFLS-4 [4,40], a similar study using data from the Indonesian health insurance program in 2015–2016, similarly, found a significant multimorbidity of hypertension with its symptoms and complications [18,40]. These contrasting data may indicate an underreporting, particularly among hypertensive patients, as hypertension may remain asymptomatic to the patient [41]. However, the lower proportion of hypertension may, instead, indicate improvements in Indonesia’s healthcare system, particularly with the introduction of the Indonesian health insurance program in 2014 [42].

Nevertheless, several findings have noted the suboptimal result of the current national program in the treatment of chronic disease patients [43], particularly in preventing comorbidities [44]. Based on our data and its comparison to the previous study from the older dataset [4], similar findings of multimorbidity prevalence should indicate the remaining inadequacy of current intervention programs in both the prevention and management of chronic diseases [45]. We believe that these issues, indicated through the prevalence of multimorbidity, corroborated multimorbid patients’ treatment nonadherence, as is further elaborated below.

### 4.2. Treatment Nonadherence among Multimorbid Patients

We found that the prevalence of nonadherence among multimorbid patients in Indonesia was largely similar to that of Ireland (23% and 31%) [46,47], Portugal (56.3%) [48], Saudi Arabia (46.5%) [49], and Spain (79.6%) [50]. However, an interventional study in Switzerland reported a lower prevalence of nonadherence; ranging between 1.5% and 14.7% [51]. A meta-analysis also found nonadherence among global multimorbid patients to an average of 42.6%, further noting the breadth of nonadherence measurement as the cause of such a range [22].

Differences in inclusion criteria and monitored characteristics render comparison among studies difficult and largely inconsistent [22]. Nonetheless, several studies have reported similar characteristics to our findings; mainly in medication regiment and complexity (by the number of morbidity) [46,47,49,51], medication knowledge and beliefs (by self-perceived health conditions) [48,52], and treatment outcome (by missing active days) [20,21,22,23]. These aspects are confirmed in one systematic review of multimorbid patients’ perspectives on medication adherence, which attributed nonadherence to perceptions of symptoms (i.e., health beliefs), economic and cultural factors (i.e., ethnicities and socioeconomic status), and failure to implement a persistent adherence [24].

Akin to global findings [53], the growing population of younger multimorbid patients should be particularly of concern. Our findings that associate treatment nonadherence with younger age may indicate poor clinical intervention among the younger population, where chronic disease and multimorbidity treatment remain to be oriented to the older population [54]. While several concerns have been raised about the increasing number of younger patients with chronic diseases [54,55], to the best of our knowledge, few studies have been dedicated to younger populations with chronic disease [56,57,58]—none were found in terms of multimorbidity and its associated treatment adherence.

This study also found that subjects with a good self-perception of health are more likely to be nonadherent; contrary to other studies reporting the association between clinical depression, multimorbidity, and medication adherence [48,59]. While this issue is often attributed to the role of social support in the management of chronic diseases, particularly associating multimorbidity itself with poorer self-perception [60,61], our result also found that living in a smaller household (of 2 to 6 members) is associated with nonadherence. In addition to the association between nonadherence to no insurance ownership and low income, we believe that the association between being in a smaller household and nonadherence was, instead, related to economic resource allocation issues [62], rather than that of social support.

Interestingly, smoking behavior was also found to be related to nonadherence. While a previous study from the United States indicated mixed results between having chronic illnesses and being an ex-smoker [63,64], we believe that smoking cessation may have indicated a part of the patient’s overall self-efficacy and behavior [65,66,67]. Corroborated by the associated current self-perceived health condition variable to nonadherence, the interpretation of smoking status as an indicator for treatment adherence, therefore, emphasized the role of knowledge in establishing treatment adherence on a personal level [68], which further implicates the need to modify smoking behavior as a part of both risk management and behavior modification at large [64].

This notion is in line with the health belief model [69], which indicates patients’ knowledge to be central to their treatment behavior [70,71]. Other studies further exemplified the role of perception in treatment behaviors [72], particularly in terms of treatment adherence [24]. We believe further consideration of patients’ experience with their chronic disease would provide potential interventions to improve their adherence [73], as is further elaborated below.

## 5. Study Implications

With particular emphasis on the distribution of nonadherence across numbers of multimorbidity, we posit that treatment adherence within the Indonesian multimorbid population is determined primarily by the patient’s treatment complexity and self-perception of health. While evidence for effective interventions to improve treatment adherence remains limited [26,74], current studies support this notion by emphasizing the need to enable patient self-efficacy through approaches such as patient-centered prescribing, as well as patient education [26,75].

In terms of treatment complexity, a metareview concluded that polypharmacy management positively impacts treatment adherence [76]. Although one systematic review indicated the lack of correlation between attempts of medication deprescribing and adherence [77], polypharmacy has generally been understood to correlate with multimorbidity and treatment nonadherence [23,78]. This notion is further supported by one interventional study which further indicated that the patient-centered prescribing model has led to a significant increase in adherence; from 22.1% to 51.9% [75].

In terms of patient education, multiple studies have correlated patients’ health beliefs and their treatment adherence [24,26]. Additionally, social support from patients’ peers was further found to be correlated with their treatment adherence [49,79]. Therefore, we argue that healthcare workers should primarily build the knowledge of patient treatment and emphasize the need for controlling chronic disease [26], with further additional education including patients’ caregivers in facilitating their adherence [80].

Noting the emphasized need for a personalized and multidisciplinary approach to improve treatment adherence [26,77,80], pharmacists are one of the healthcare workers serving a crucial role in ensuring patients’ effective treatment through medication counseling and management [74,75,81], in accordance with patients’ needs [80]. Therefore, we propose an intervention to emphasize patients’ expectations and responsibilities of their treatment [82,83], i.e., that chronic disease treatment is meant to manage and prevent its complications rather than “curing” it [84,85]. We believe the adaptation of such interventions may improve medication adherence in Indonesia.

## 6. Strengths and Limitations

To the best of our knowledge, this study is the first to identify issues in treatment adherence among multimorbid patients using big data in Indonesia. The use of survey data constitutes a high representability of the findings to the general Indonesian population. Furthermore, the use of standardized reporting guidelines in this study should allow systematic and transparent reporting of the results.

However, the survey-based data of IFLS-5 may pose recall and social desirability biases. The cross-sectional nature of this study should also be cautiously interpreted for the causality between factors and outcomes. The limited data in this study also prohibits analysis of other factors that may have contributed as factors to treatment adherence.

## 7. Conclusions

Multimorbidity presents a unique challenge to patients’ treatment adherence. While the factors associated with multimorbidity have generally been well known from previous studies, findings of nonadherence in our study indicate the suboptimal interventions to multimorbid patients. Based on this finding of various nonadherence characteristics, we highlight the need for healthcare workers to tailor their intervention based on individual problems related to treatment adherence through approaches such as medication management and patient education. The targeted intervention should be based on the individual patient’s problems and characteristics to improve treatment adherence.

## Figures and Tables

**Table 1:** Prevalence of multimorbidity combinations (*n* = 3513).

Order	Disease Dyads	*n* (%)	Disease Triads	*n* (%)
1	Hypertension—Digestive disease	185 (14.5%)	Hypertension—Digestive disease—Arthritis	20 (1.6%)
2	Hypertension—Arthritis	118 (9.2%)	Hypertension—Digestive disease—High cholesterol	17 (1.3%)
3	Hypertension—High cholesterol	75 (5.9%)	Hypertension—Arthritis—High cholesterol	12 (0.9%)
4	Arthritis—Digestive disease	55 (4.3%)	Hypertension—Arthritis—Asthma	6 (0.5%)
5	Digestive disease—Asthma	47 (3.7%)	Digestive disease—Asthma—Other lung disease	6 (0.5%)

**Table 2:** Characteristics of included subjects (*n* = 3513).

No.	Characteristics			Proportion (%)
1.	Socioeconomic-related factors			
		Female sex *		61.7%
		Age (years)		
			15–65	81.0%
			>65	19.0%
		Obtained formal education *		91.7%
		Non-Javanese ethnicity *		57.5%
		Non-Java residence *		43.1%
		Rural residence *		34.0%
		Household size (people)		
			1	2.0%
			2–6	58.6%
			>6	39.5%
		Annual income (IDR) *		
			Not working	39.6%
			<40 million	51.4%
			40–100 million	6.9%
			>100 million	1.4%
		No health insurance ownership *		42.1%
2.	Patient-related factors			
		Healthy current self-perceived health status		50.2%
		Healthy future self-perceived health status *		76.9%
		≤7 days of missing active days in the last month *		82.1%
		No depressive symptoms *		61.9%
3.	Disease-related factors			
		Body mass index *		
			Obese	15.0%
			Overweight	30.0%
			Normal	39.4%
			Underweight	8.9%
		Smoking behavior		
			Ex-smoker	11.7%
			Non-smoker	67.5%
			Active smoker	20.8%
4.	Nonadherent subjects			36.4%

* Missing value = Sex 1 (0.0%), Education 29 (0.8%), Ethnicity 18 (0.5%), Demographic residence 1 (0.0%), Geographic residence 1 (0.0%), Annual income 24 (0.7%), Health insurance ownership 14 (0.4%), Future self-perceived health status 441 (12.6%), Missing active days 3 (0.1%), BMI 235 (6.7%), Depressive symptoms 432 (12.3%). In 2014–2015, USD 1 was equivalent, on average, to IDR 13,118.

**Table 3:** Characteristics associated with treatment nonadherence (*n* = 3513).

No.	Characteristics	Adherent (*n* = 2236)	Nonadherent (*n* = 1277)	Bivariate		Multivariate	
				OR (95% CI)	*p*-Value	aOR (95% CI)	*p*-Value
1	Socioeconomic factors						
	Female	1402 (64.7%)	766 (35.3%)	0.89 (0.77–1.03)	0.107 *	0.87 (0.73–1.10)	0.303
	Age (years)						
	15–65	1752 (61.5)	1095 (38.5%)	1.66 (1.38–2.00)	0.000 *	1.25 (1.01–1.55)	0.045 **
	>65	484 (72.7%)	182 (27.3%)	Ref			
	Obtained formal education	2038 (63.3%)	1183 (36.7%)	1.30 (0.99–1.71)	0.055 *	1.02 (0.76–1.36)	0.901
	Non-Javanese ethnicity	1277 (63.2%)	743 (36.8%)	1.05 (0.92–1.21)	0.470	N/A	
	Non-Java residence	948 (62.7%)	565 (37.3%)	1.08 (0.94–1.24)	0.293	N/A	
	Rural residence	752 (62.9%)	443 (37.1%)	1.05 (0.91–1.21)	0.530	N/A	
	Household size (people)						
	1	44 (62.9%)	26 (37.1%)	1.23 (0.75–2.03)	0.409	1.02 (0.62–1.70)	0.930
	2–6	1255 (61.0%)	802 (39.0%)	1.33 (1.16–1.54)	0.000 *	1.17 (1.01–1.36)	0.046 **
	>6	937 (67.6%)	449 (32.4%)	Ref			
	Annual income (IDR)						
	Not working	959 (68.9%)	432 (31.1%)	Ref			
	<40 million	1085 (60.1%)	721 (39.9%)	1.48 (1.27–1.71)	0.000 *	1.23 (1.04–1.46)	0.015 **
	40–100 million	153 (62.7%)	91 (37.3%)	1.32 (1.00–1.75)	0.054 *	1.00 (0.73–1.36)	0.982
	>100 million	25 (52.1%)	23 (47.9%)	2.04 (1.15–3.64)	0.015 *	1.52 (0.83–2.77)	0.174
	No health insurance ownership	915 (61.9%)	564 (38.1%)	1.14 (0.99–1.30)	0.075 *	1.20 (1.04–1.39)	0.015 **
2	Patient-related factors						
	Healthy current self-perceived health status	985 (55.8%)	780 (44.2%)	1.99 (1.73–2.29)	0.000 *	1.79 (1.54–2.08)	0.000 **
	Healthy future self-perceived health status	N/A					
	≤7 days of missing active days in the last month	1763 (61.2%)	1120 (38.8%)	1.90 (1.56–2.31)	0.000 *	1.36 (1.10–1.68)	0.005 **
	No depressive symptoms	N/A					
3	Condition-related factors						
	Body mass index						
	Obese	347 (65.8%)	180 (34.2%)	Ref		N/A	
	Overweight	682 (64.8%)	371 (35.2%)	1.05 (0.84–1.31)	0.672		
	Normal	862 (62.3%)	522 (37.7%)	1.17 (0.95–1.44)	0.149		
	Underweight	192 (61.1%)	122 (38.9%)	1.23 (0.92–1.64)	0.170		
	Smoking behavior						
	Ex-smoker	300 (72.8%)	112 (27.2%)	Ref			
	Non-smoker	1510 (63.7%)	861 (36.3%)	1.53 (1.21–1.93)	0.000 *	1.44 (1.08–1.90)	0.012 **
	Active smoker	426 (58.4%)	304 (41.6%)	1.91 (1.47–2.49)	0.000 *	1.51 (1.14–1.99)	0.004 **

* = Bivariate analysis statistically significant using *p* < 0.25%; ** = multivariate analysis statistically significant using *p* < 0.05; Ref = reference; N/A = not included. The multivariate model was validated using the Hosmer–Lemeshow test (χ^2^ = 7.306 and *p* = 0.504). In 2014–2015, USD 1 was equivalent, on average, to IDR 13,118.

## Data Availability

The IFLS-5 data are available through a request directed to RAND Corporation at https://www.rand.org/well-being/social-and-behavioral-policy/data/FLS/IFLS/access.html. We accessed this data on 27 May 2023.

## Supplementary Materials

The following supporting information can be downloaded at: https://www.mdpi.com/article/10.3390/medicina60040634/s1, Table S1: Operational definition of variables; Table S2: STROBE statement checklist for cross-sectional studies.

## Abbreviations

95% CI	95% confidence interval
aOR	Adjusted odds ratio
BMI	Body mass index
IFLS	Indonesian Family Life Survey project: A longitudinal household survey initiated by RAND Corporation
IDR	Indonesian Rupiah
IFLS-4	The fourth Indonesian Family Life Survey, conducted in 2007/2008
IFLS-5	The fifth Indonesian Family Life Survey, conducted in 2014/2015
OR	Odds ratio
STROBE	Strengthening the Reporting of Observational Studies in Epidemiology
USD	United States Dollar

## References

[1] Academy of Medical Sciences (2018). Multimorbidity: A Priority for Global Health Research.

[2] Siswati T., Paramashanti B.A., Rialihanto M.P., Waris L. (2022). Epidemiological Transition in Indonesia and Its Prevention: A Narrative Review. J. Complement. Altern. Med. Res..

[3] Vos T., Lim S.S., Abbafati C., Abbas K.M., Abbasi M., Abbasifard M., Abbasi-Kangevari M., Abbastabar H., Abd-Allah F., Abdelalim A. (2020). Global Burden of 369 Diseases and Injuries in 204 Countries and Territories, 1990–2019: A Systematic Analysis for the Global Burden of Disease Study 2019. Lancet.

[4] Hussain M.A., Huxley R.R., Al Mamun A. (2015). Multimorbidity Prevalence and Pattern in Indonesian Adults: An Exploratory Study Using National Survey Data. BMJ Open.

[5] World Health Organization (2021). Political Declaration of the Third High-Level Meeting of the General Assembly on the Prevention and Control of Non-Communicable Diseases.

[6] Wolff J.L., Starfield B., Anderson G. (2002). Prevalence, Expenditures, and Complications of Multiple Chronic Conditions in the Elderly. Arch. Intern. Med..

[7] Calderón-Larrañaga A., Poblador-Plou B., González-Rubio F., Gimeno-Feliu L.A., Abad-Díez J.M., Prados-Torres A. (2012). Multimorbidity, Polypharmacy, Referrals, and Adverse Drug Events: Are We Doing Things Well?. Br. J. Gen. Pract..

[8] Marengoni A., Onder G. (2015). Guidelines, Polypharmacy, and Drug-Drug Interactions in Patients with Multimorbidity. BMJ.

[9] Kadam U., Croft P. (2007). North Staffordshire GP Consortium Group Clinical Multimorbidity and Physical Function in Older Adults: A Record and Health Status Linkage Study in General Practice. Fam. Pract..

[10] Arokiasamy P., Uttamacharya U., Jain K., Biritwum R.B., Yawson A.E., Wu F., Guo Y., Maximova T., Espinoza B.M., Salinas Rodríguez A. (2015). The Impact of Multimorbidity on Adult Physical and Mental Health in Low- and Middle-Income Countries: What Does the Study on Global Ageing and Adult Health (SAGE) Reveal?. BMC Med..

[11] Ramond-Roquin A., Haggerty J., Lambert M., Almirall J., Fortin M. (2016). Different Multimorbidity Measures Result in Varying Estimated Levels of Physical Quality of Life in Individuals with Multimorbidity: A Cross-Sectional Study in the General Population. BioMed Res. Int..

[12] Wei M.Y., Kawachi I., Okereke O.I., Mukamal K.J. (2016). Diverse Cumulative Impact of Chronic Diseases on Physical Health–Related Quality of Life: Implications for a Measure of Multimorbidity. Am. J. Epidemiol..

[13] Marthias T., Anindya K., Ng N., McPake B., Atun R., Arfyanto H., Hulse E.S., Zhao Y., Jusril H., Pan T. (2021). Impact of Non-Communicable Disease Multimorbidity on Health Service Use, Catastrophic Health Expenditure and Productivity Loss in Indonesia: A Population-Based Panel Data Analysis Study. BMJ Open.

[14] Wang L., Si L., Cocker F., Palmer A.J., Sanderson K. (2018). A Systematic Review of Cost-of-Illness Studies of Multimorbidity. Appl. Health Econ. Health Policy.

[15] Sum G., Hone T., Atun R., Millett C., Suhrcke M., Mahal A., Koh G.C.-H., Lee J.T. (2018). Multimorbidity and Out-of-Pocket Expenditure on Medicines: A Systematic Review. BMJ Glob. Health.

[16] Anindya K., Ng N., Atun R., Marthias T., Zhao Y., McPake B., Van Heusden A., Pan T., Lee J.T. (2021). Effect of Multimorbidity on Utilisation and Out-of-Pocket Expenditure in Indonesia: Quantile Regression Analysis. BMC Health Serv. Res..

[17] Tran P.B., Kazibwe J., Nikolaidis G.F., Linnosmaa I., Rijken M., Van Olmen J. (2022). Costs of Multimorbidity: A Systematic Review and Meta-Analyses. BMC Med..

[18] Husnayain A., Ekadinata N., Sulistiawan D., Chia-Yu Su E. (2020). Multimorbidity Patterns of Chronic Diseases among Indonesians: Insights from Indonesian National Health Insurance (INHI) Sample Data. Int. J. Environ. Res. Public Health.

[19] Sabaté E. (2003). Adherence to Long-Term Therapies: Evidence for Action.

[20] Walsh C.A., Cahir C., Tecklenborg S., Byrne C., Culbertson M.A., Bennett K.E. (2019). The Association between Medication Non-adherence and Adverse Health Outcomes in Ageing Populations: A Systematic Review and Meta-analysis. Br. J. Clin. Pharmacol..

[21] Cutler R.L., Fernandez-Llimos F., Frommer M., Benrimoj C., Garcia-Cardenas V. (2018). Economic Impact of Medication Non-Adherence by Disease Groups: A Systematic Review. BMJ Open.

[22] Foley L., Larkin J., Lombard-Vance R., Murphy A.W., Hynes L., Galvin E., Molloy G.J. (2021). Prevalence and Predictors of Medication Non-Adherence among People Living with Multimorbidity: A Systematic Review and Meta-Analysis. BMJ Open.

[23] Zelko E., KlemencKetis Z., TusekBunc K. (2016). Medication Adherence in Elderly with Polypharmacy Living at Home: A Systematic Review of Existing Studies. Mater. Socio Medica.

[24] Maffoni M., Traversoni S., Costa E., Midão L., Kardas P., Kurczewska-Michalak M., Giardini A. (2020). Medication Adherence in the Older Adults with Chronic Multimorbidity: A Systematic Review of Qualitative Studies on Patient’s Experience. Eur. Geriatr. Med..

[25] World Health Organization (2016). Multimorbidity.

[26] Williams A., Manias E., Walker R. (2008). Interventions to Improve Medication Adherence in People with Multiple Chronic Conditions: A Systematic Review. J. Adv. Nurs..

[27] RAND Corporation The Indonesian Family Life Survey. https://www.rand.org/well-being/social-and-behavioral-policy/data/FLS/IFLS.html.

[28] Strauss J., Witoelar F., Sikoki B. (2016). The Fifth Wave of the Indonesia Family Life Survey: Overview and Field Report: Volume 1.

[29] Strauss J., Witoelar F., Sikoki B. (2016). User’s Guide for the Indonesia Family Life Survey, Wave 5: Volume 2.

[30] Radloff L.S. (1977). The CES-D Scale: A Self-Report Depression Scale for Research in the General Population. Appl. Psychol. Meas..

[31] National Institutes of Health (2013). Managing Overweight and Obesity in Adults: Systematic Evidence Review from the Expert Panel.

[32] Madley-Dowd P., Hughes R., Tilling K., Heron J. (2019). The Proportion of Missing Data Should Not Be Used to Guide Decisions on Multiple Imputation. J. Clin. Epidemiol..

[33] IBM Corporation (2013). IBM SPSS Statistics for Windows.

[34] Von Elm E., Altman D.G., Egger M., Pocock S.J., Gøtzsche P.C., Vandenbroucke J.P. (2008). The Strengthening the Reporting of Observational Studies in Epidemiology (STROBE) Statement: Guidelines for Reporting Observational Studies. J. Clin. Epidemiol..

[35] Muggah E., Graves E., Bennett C., Manuel D.G. (2013). Ascertainment of Chronic Diseases Using Population Health Data: A Comparison of Health Administrative Data and Patient Self-Report. BMC Public Health.

[36] Kriegsman D.M.W., Penninx B.W.J.H., Van Eijk J.T.M., Boeke A.J.P., Deeg D.J.H. (1996). Self-Reports and General Practitioner Information on the Presence of Chronic Diseases in Community Dwelling Elderly. J. Clin. Epidemiol..

[37] Frost M., Wraae K., Gudex C., Nielsen T., Brixen K., Hagen C., Andersen M. (2012). Chronic Diseases in Elderly Men: Underreporting and Underdiagnosis. Age Ageing.

[38] Nguyen H., Manolova G., Daskalopoulou C., Vitoratou S., Prince M., Prina A.M. (2019). Prevalence of Multimorbidity in Community Settings: A Systematic Review and Meta-Analysis of Observational Studies. J. Comorbidity.

[39] Chowdhury S.R., Chandra Das D., Sunna T.C., Beyene J., Hossain A. (2023). Global and Regional Prevalence of Multimorbidity in the Adult Population in Community Settings: A Systematic Review and Meta-Analysis. eClinicalMedicine.

[40] Kokubo Y., Iwashima Y. (2015). Higher Blood Pressure as a Risk Factor for Diseases Other Than Stroke and Ischemic Heart Disease. Hypertension.

[41] Levy P.D., Cline D. (2009). Asymptomatic Hypertension in the Emergency Department: A Matter of Critical Public Health Importance. Acad. Emerg. Med..

[42] Agustina R., Dartanto T., Sitompul R., Susiloretni K.A., Achadi E.L., Taher A., Wirawan F., Sungkar S., Sudarmono P., Suparmi (2019). Universal Health Coverage in Indonesia: Concept, Progress, and Challenges. Lancet.

[43] Alkaff F.F., Illavi F., Salamah S., Setiyawati W., Ramadhani R., Purwantini E., Tahapary D.L. (2021). The Impact of the Indonesian Chronic Disease Management Program (PROLANIS) on Metabolic Control and Renal Function of Type 2 Diabetes Mellitus Patients in Primary Care Setting. J. Prim. Care Community Health.

[44] Alkaff F.F., Sukmajaya W.P., Intan R.E., Salamah S. (2020). Effectivity of Indonesia Chronic Disease Management Program (PROLANIS) to Control Hypertension and Its Comorbidities at Primary Health Care: Effectivity of PROLANIS to Control Hypertension. Open Access Maced. J. Med. Sci..

[45] Schröders J., Wall S., Hakimi M., Dewi F.S.T., Weinehall L., Nichter M., Nilsson M., Kusnanto H., Rahajeng E., Ng N. (2017). How Is Indonesia Coping with Its Epidemic of Chronic Noncommunicable Diseases? A Systematic Review with Meta-Analysis. PLoS ONE.

[46] Walsh C.A., Bennett K.E., Wallace E., Cahir C. (2020). Identifying Adherence Patterns Across Multiple Medications and Their Association with Health Outcomes in Older Community-Dwelling Adults with Multimorbidity. Value Health.

[47] Kim S., Bennett K., Wallace E., Fahey T., Cahir C. (2018). Measuring Medication Adherence in Older Community-Dwelling Patients with Multimorbidity. Eur. J. Clin. Pharmacol..

[48] Félix I.B., Henriques A. (2021). Medication Adherence and Related Determinants in Older People with Multimorbidity: A Cross-sectional Study. Nurs. Forum.

[49] Almutairi A.S., Alhazmi T.M., Alotaibi Y.H., Alfraidi A.A., Alsaad A.M., Matrood R.A., Al-khatir A.N., Alsubaie A.A., Alotibi W.M. (2022). Medication Adherence Among Multimorbid Patients with Polypharmacy and Its Relation to Social Support at National Guard Primary Health Care Centers, Riyadh. Cureus.

[50] González-Bueno J., Sevilla-Sánchez D., Puigoriol-Juvanteny E., Molist-Brunet N., Codina-Jané C., Espaulella-Panicot J. (2021). Factors Associated with Medication Non-Adherence among Patients with Multimorbidity and Polypharmacy Admitted to an Intermediate Care Center. Int. J. Environ. Res. Public Health.

[51] Inauen J., Bierbauer W., Lüscher J., König C., Tobias R., Ihle A., Zimmerli L., Holzer B.M., Battegay E., Siebenhüner K. (2017). Assessing Adherence to Multiple Medications and in Daily Life among Patients with Multimorbidity. Psychol. Health.

[52] Schüz B., Marx C., Wurm S., Warner L.M., Ziegelmann J.P., Schwarzer R., Tesch-Römer C. (2011). Medication Beliefs Predict Medication Adherence in Older Adults with Multiple Illnesses. J. Psychosom. Res..

[53] Afshar S., Roderick P.J., Kowal P., Dimitrov B.D., Hill A.G. (2015). Multimorbidity and the Inequalities of Global Ageing: A Cross-Sectional Study of 28 Countries Using the World Health Surveys. BMC Public Health.

[54] Van Den Akker M., Dieckelmann M., Hussain M.A., Bond-Smith D., Muth C., Pati S., Saxena S., Silva D., Skoss R., Straker L. (2022). Children and Adolescents Are Not Small Adults: Toward a Better Understanding of Multimorbidity in Younger Populations. J. Clin. Epidemiol..

[55] Whitty C.J.M., MacEwen C., Goddard A., Alderson D., Marshall M., Calderwood C., Atherton F., McBride M., Atherton J., Stokes-Lampard H. (2020). Rising to the Challenge of Multimorbidity. BMJ.

[56] Pai A., Ostendorf H.M. (2011). Treatment Adherence in Adolescents and Young Adults Affected by Chronic Illness during the Health Care Transition from Pediatric to Adult Health Care: A Literature Review. Child. Health Care.

[57] Sawyer S.M., Drew S., Yeo M.S., Britto M.T. (2007). Adolescents with a Chronic Condition: Challenges Living, Challenges Treating. Lancet.

[58] Venning A., Eliott J., Wilson A., Kettler L. (2008). Understanding Young Peoples’ Experience of Chronic Illness: A Systematic Review. Int. J. Evid. Based Healthc..

[59] Sinaga I.O.Y., Barliana M.I., Pradipta I.S., Iskandarsyah A., Abdulah R., Alfian S.D. (2022). Depression Is Associated with the Increase Risk of Multimorbidity Among the General Population in Indonesia. J. Multidiscip. Healthc..

[60] Fortin M. (2006). Psychological Distress and Multimorbidity in Primary Care. Ann. Fam. Med..

[61] Cavalcanti G., Doring M., Portella M.R., Bortoluzzi E.C., Mascarelo A., Dellani M.P. (2017). Multimorbidity Associated with Polypharmacy and Negative Self-Perception of Health. Rev. Bras. Geriatr. E Gerontol..

[62] Kolling M., Winkley K., Von Deden M. (2010). “For Someone Who’s Rich, It’s Not a Problem”. Insights from Tanzania on Diabetes Health-Seeking and Medical Pluralism among Dar Es Salaam’s Urban Poor. Glob. Health.

[63] Patel K., Schlundt D., Larson C., Wang H., Brown A., Hargreaves M. (2009). Chronic Illness and Smoking Cessation. Nicotine Tob. Res..

[64] Kalkhoran S., Kruse G.R., Chang Y., Rigotti N.A. (2018). Smoking-Cessation Efforts by US Adult Smokers with Medical Comorbidities. Am. J. Med..

[65] Gwaltney C.J., Metrik J., Kahler C.W., Shiffman S. (2009). Self-Efficacy and Smoking Cessation: A Meta-Analysis. Psychol. Addict. Behav..

[66] Martinez E., Tatum K.L., Glass M., Bernath A., Ferris D., Reynolds P., Schnoll R.A. (2010). Correlates of Smoking Cessation Self-Efficacy in a Community Sample of Smokers. Addict. Behav..

[67] Galvin K.T. (1992). A Critical Review of the Health Belief Model in Relation to Cigarette Smoking Behaviour. J. Clin. Nurs..

[68] Friis K., Lasgaard M., Pedersen M.H., Duncan P., Maindal H.T. (2019). Health Literacy, Multimorbidity, and Patient-Perceived Treatment Burden in Individuals with Cardiovascular Disease. A Danish Population-Based Study. Patient Educ. Couns..

[69] Rosenstock I.M. (1974). The Health Belief Model and Preventive Health Behavior. Health Educ. Monogr..

[70] Wang T., Wang H., Zeng Y., Cai X., Xie L. (2022). Health Beliefs Associated with Preventive Behaviors against Noncommunicable Diseases. Patient Educ. Couns..

[71] Widayanti A.W., Green J.A., Heydon S., Norris P. (2020). Health-Seeking Behavior of People in Indonesia: A Narrative Review. J. Epidemiol. Glob. Health.

[72] Foley L., Hynes L., Murphy A.W., Molloy G.J. (2022). ‘Just Keep Taking Them, Keep Hoping They’Ll Work’: A Qualitative Study of Adhering to Medications for Multimorbidity. Br. J. Health Psychol..

[73] Duguay C., Gallagher F., Fortin M. (2014). The Experience of Adults with Multimorbidity: A Qualitative Study. J. Comorbidity.

[74] Yang C., Zhu S., Lee D.T.F., Chair S.Y. (2022). Interventions for Improving Medication Adherence in Community-Dwelling Older People with Multimorbidity: A Systematic Review and Meta-Analysis. Int. J. Nurs. Stud..

[75] González-Bueno J., Sevilla-Sánchez D., Puigoriol-Juvanteny E., Molist-Brunet N., Codina-Jané C., Espaulella-Panicot J. (2022). Improving Medication Adherence and Effective Prescribing through a Patient-Centered Prescription Model in Patients with Multimorbidity. Eur. J. Clin. Pharmacol..

[76] Ali M.U., Sherifali D., Fitzpatrick-Lewis D., Kenny M., Lamarche L., Raina P., Mangin D. (2022). Interventions to Address Polypharmacy in Older Adults Living with Multimorbidity: Review of Reviews. Can. Fam. Physician.

[77] Ulley J., Harrop D., Ali A., Alton S., Fowler Davis S. (2019). Deprescribing Interventions and Their Impact on Medication Adherence in Community-Dwelling Older Adults with Polypharmacy: A Systematic Review. BMC Geriatr..

[78] Payne R.A., Avery A.J., Duerden M., Saunders C.L., Simpson C.R., Abel G.A. (2014). Prevalence of Polypharmacy in a Scottish Primary Care Population. Eur. J. Clin. Pharmacol..

[79] Lozano-Hernández C.M., López-Rodríguez J.A., Leiva-Fernández F., Calderón-Larrañaga A., Barrio-Cortes J., Gimeno-Feliu L.A., Poblador-Plou B., Cura-González I.D., MULTIPAP GROUP (2020). Social Support, Social Context and Nonadherence to Treatment in Young Senior Patients with Multimorbidity and Polypharmacy Followed-up in Primary Care. MULTIPAP Study. PLoS ONE.

[80] Giardini A., Maffoni M., Kardas P., Costa E. (2018). A Cornerstone of Healthy Aging: Do We Need to Rethink the Concept of Adherence in the Elderly?. Patient Prefer. Adherence.

[81] Vasilevskis E.E., Shah A.S., Hollingsworth E.K., Shotwell M.S., Mixon A.S., Bell S.P., Kripalani S., Schnelle J.F., Simmons S.F., The Shed-MEDS Team (2019). A Patient-Centered Deprescribing Intervention for Hospitalized Older Patients with Polypharmacy: Rationale and Design of the Shed-MEDS Randomized Controlled Trial. BMC Health Serv. Res..

[82] Laferton J.A.C., Kube T., Salzmann S., Auer C.J., Shedden-Mora M.C. (2017). Patients’ Expectations Regarding Medical Treatment: A Critical Review of Concepts and Their Assessment. Front. Psychol..

[83] Farley H. (2020). Promoting Self-efficacy in Patients with Chronic Disease beyond Traditional Education: A Literature Review. Nurs. Open.

[84] Dwarswaard J., Bakker E.J.M., Van Staa A., Boeije H.R. (2016). Self-management Support from the Perspective of Patients with a Chronic Condition: A Thematic Synthesis of Qualitative Studies. Health Expect..

[85] Lawn S., McMillan J., Pulvirenti M. (2011). Chronic Condition Self-Management: Expectations of Responsibility. Patient Educ. Couns..

[86] RAND Corporation IFLS Project Teams and Funding. https://www.rand.org/well-being/social-and-behavioral-policy/data/FLS/IFLS/teamfund.html.

[87] WMA General Assembly (2013). WMA Declaration of Helsinki—Ethical Principles for Medical Research Involving Human Subjects.

