# Supplementary material for: Treatment Nonadherence among Multimorbid Chronic Disease Patients: Evidence from 3515 Subjects in Indonesia
Source: Medicina (Kaunas). 2024 Apr 15;60(4):634. doi: 10.3390/medicina60040634 (PMC11052292; doi:10.3390/medicina60040634)
Supplement: Supplementary file 1 [file medicina-60-00634-s001.zip › medicina-2943955-supplementary.pdf]

Supplementary materials

Treatment Nonadherence among Multimorbid Chronic Disease Patients: Evidence from 3,515 Subjects in Indonesia

Ivan Surya Pradipta, Kevin Aprilio, Yozi Fiedya Ningsih, Mochammad Andhika Aji Pratama, Sofa Dewi Alfian, Rizky Abdulah

Table S1. Operational definition of variables

No.	Variables	Definition
**Exposure**		
1.	Sex	Biological characteristic of subjects. This variable was classified into male and female.
2.	Age	Age of subjects during the IFLS-5 study. In this study, this variable was classified into < 15 years, 15-65 years, and > 65 years.
3.	Formal education	Subject's completion of any formal education based on Indonesian education system. In this study, this variable was classified into schooled and unschooled.
4.	Ethnicity	Subject's self-identified ethnicity identity. In this study, this variable was classified into Javanese and non-Javanese.
5.	Geographical residence	Geographical location of subject's residence during the IFLS-5 study. This variable was measured by subject's provincial location of residence and classified in this study into Java and non-Java residence.
6.	Demographical residence	Demographical condition of the subject's residence during the IFLS-5 study. This variable was classified into rural and urban residence.
7.	Household size	Total number of household members in a subject's residence. In this study, this variable was classified into 1 person (i.e., living alone), 2-6 people, and > 6 people.

8.	Annual income	Total annual earnings of each subject in Indonesian Rupiah (IDR). In this study, this variable was classified into not working (i.e., having no income), < 40 million, 40-100 million, and > 100 million.
9.	Health insurance ownership	Subject's participation or ownership of public and/or private health insurance program/policy.
10.	Current self-perceived health status	Subject's observation of their health condition during the IFLS-5 study. This variable was measured with the question *"In general, how is your health?"*, with responses being very healthy; somewhat healthy; somewhat unhealthy; and very unhealthy. In this study, this variable was classified to unhealthy (somewhat unhealthy and very unhealthy) and healthy (very healthy and somewhat healthy).
	Future self-perceived health status	Subject's observation of their health condition during the IFLS-5 study. This variable was measured with the question *"In general, how would you expect your health in the next year compared to now?"*, with responses being very healthier; somewhat healtier; about the same; somewhat unhealthier; and very unhealthier. In this study, this variable was classified to unhealthy (somewhat unhealthier and very unhealthier) and healthy (about the same, somewhat healthier, and very healthier).
11.	Active days missed in the last month	The number of days in the last month of the IFLS-5 study in which a subject misses their primary daily activities due to poor health. This variable was measured with the question *"During the last 4 weeks, how many days of your primary activities did you miss due to poor health?"*, with responses classified in this study into ≤ 7 days and > 7 days.
12.	Depressive symptoms	Subject's symptoms of depression aside from clinical diagnosis. This variable was measured using the Center for Epidemiologic Studies Depression Scale (CES-D) questionnaire. This variable was classified into not having depressive symptoms and having depressive symptoms.
13.	Body mass index	Calculation of subject's body mass index (BMI). Subject's height and weight was measured with Seca plastic height board 213 to the

		nearest milimeter and Camry EB1003 to the nearest tenth of a kilogram, respectively. This variable was classified into obese, overweight, normal, and underweight BMI.
14.	Smoking behavior	Tobacco consumption behavior of subject prior to and during the IFLS-5 study. This variable was measured with two questions: *“Have you ever chewed tobacco, smoked a pipe, smoked self-rolled cigarettes, or smoked cigarettes/cigars?”* and *“Do you still have the habit or have you totally quit?”*, with responses classified in this study into non-smoker; ex-smoker; and active smoker.
**Outcome**		
15.	Treatment nonadherence	Subject’s treatment for each chronic disease, including: hypertension, digestive diseases, arthritis, hypercholesterolemia, diabetes mellitus, asthma, cardiovascular diseases, other lung diseases, kidney diseases, stroke, tuberculosis, liver diseases, cancer or other malignancies, prostate disease, memory-related diseases, and psychiatric diseases.<br><br>This variable was measured with a question *“Are you taking [types of treatment] to treat [types of chronic disease] and its complications?”*. Subjects reporting no treatment for any of the chronic diseases were classified in this study as nonadherent.

Table S2. STROBE statement checklist for cross-sectional studies

**Section**	**Item no.**	**Recommendation**	**Page no.**
**Title and abstract**			
	1.	(a) Indicate the study’s design with a commonly used term in the title or the abstract	1
		(b) Provide in the abstract an informative and balanced summary of what was done and what was found	1
**Introduction**			
Background or	2.	Explain the scientific background and rationale for the	1-2

rationale		investigation being reported	
Objectives	3.	State specific objectives, including any prespecified hypotheses	1-2
**Methods**			
Study design	4.	Present key elements of study design early in the paper	2-3
Setting	5.	Describe the setting, locations, and relevant dates, including periods of recruitment, exposure, follow-up, and data collection.	2
Participants	6.	Give the eligibility criteria, and the sources and methods of selection for participants	2-3
Variables	7.	Clearly define all outcomes, exposures, predictors, potential confounders, and effect modifiers. Give diagnostic criteria, if applicable.	2-3<br>Table S1*
Data sources or measurement	8.	For each variable of interest, give sources of data and details of methods of assessment (measurement). Describe comparability of assessment methods of there is more than one group.	Table S1*
Bias	9.	Describe any efforts to address potential sources of bias	N/A
Study size	10.	Explain how the study size was arrived at	3-4<br>Figure 1
Quantitative variables	11.	Explain how quantitative variables were handled in the analyses. If applicable, describe which groupings were chosen and why	Table S1*
Statistical methods	12.	(a) Describe all statistical methods, including those used to control for confounding	3
		(b) Describe any methods used to examine subgroups and interactions	N/A
		(c) Explain how missing data were addressed	3
		(d) If applicable, describe analytical methods taking account of sampling strategy	N/A

		(e) Describe any sensitivity analyses	N/A
**Results**			
Participants	13.	(a) Report numbers of individuals at each stage of study—eg numbers potentially eligible, examined for eligibility, confirmed eligible, included in the study, completing follow-up, and analysed	3-4<br>Figure 1
		(b) Give reasons for non-participation at each stage	3-4<br>Figure 1
		(c) Consider use of a flow diagram	Figure 1
Descriptive data	14.	(a) Give characteristics of study participants (eg demographic, clinical, social) and information on exposures and potential confounders	3-6<br>Figure 2<br>Table 1<br>Table 2
		(b) Indicate number of participants with missing data for each variable of interest	5-6<br>Table 2
Outcome data	15.	Report numbers of outcome events or summary measures	3-6<br>Figure 2<br>Table 2
Main results	16.	(a) Give unadjusted estimates and, if applicable, confounder-adjusted estimates and their precision (eg, 95% confidence interval). Make clear which confounders were adjusted for and why they were included.	6-7<br>Table 3
		(b) Report category boundaries when continuous variables were categorized	Table S1*
		(c) If relevant, consider translating estimates of relative risk into absolute risk for a meaningful time period	N/A
Other analyses	17.	Report other analyses done—eg analyses of subgroup interactions, and sensitivity analyses	N/A
**Discussion**			

Key results	18.	Summarize key results with reference to study objectives	7
Limitation	19.	Discuss limitations of the study, taking into account sources of potential bias or imprecision. Discuss both direction and magnitude of any potential bias	7
	20.	Give a cautious overall interpretation of results considering objectives, limitations, multiplicity of analyses, results from similar studies, and other relevant evidence	7-10
Generalizability	21.	Discuss the generalizability (external validity) of the study results	9-10
Other information			
Funding	22.	Give the source of funding and the role of the funders for the present study and, if applicable, for the original study on which the present article is based.	10

Note: * = sources from supplementary materials; N/A = not applicable or conducted.
